# Systematic identification of transcriptional regulatory modules from protein–protein interaction networks

**DOI:** 10.1093/nar/gkt913

**Published:** 2013-10-16

**Authors:** Diego Diez, Andrew Paul Hutchins, Diego Miranda-Saavedra

**Affiliations:** ^1^World Premier International (WPI) Immunology Frontier Research Center (IFReC), Osaka University, 3-1 Yamadaoka, Suita 565-0871, Osaka, Japan, ^2^South China Institute for Stem Cell Biology and Regenerative Medicine, Guangzhou Institutes of Biomedicine and Health, Chinese Academy of Sciences, 190 Kaiyuan Ave, Guangzhou 510663, China and ^3^Fibrosis Laboratories, Institute of Cellular Medicine, Newcastle University Medical School, Framlington Place, Newcastle upon Tyne NE2 4HH, United Kingdom

## Abstract

Transcription factors (TFs) combine with co-factors to form transcriptional regulatory modules (TRMs) that regulate gene expression programs with spatiotemporal specificity. Here we present a novel and generic method (rTRM) for the reconstruction of TRMs that integrates genomic information from TF binding, cell type-specific gene expression and protein–protein interactions. rTRM was applied to reconstruct the TRMs specific for embryonic stem cells (ESC) and hematopoietic stem cells (HSC), neural progenitor cells, trophoblast stem cells and distinct types of terminally differentiated CD4^+^ T cells. The ESC and HSC TRM predictions were highly precise, yielding 77 and 96 proteins, of which ∼75% have been independently shown to be involved in the regulation of these cell types. Furthermore, rTRM successfully identified a large number of bridging proteins with known roles in ESCs and HSCs, which could not have been identified using genomic approaches alone, as they lack the ability to bind specific DNA sequences. This highlights the advantage of rTRM over other methods that ignore PPI information, as proteins need to interact with other proteins to form complexes and perform specific functions. The prediction and experimental validation of the co-factors that endow master regulatory TFs with the capacity to select specific genomic sites, modulate the local epigenetic profile and integrate multiple signals will provide important mechanistic insights not only into how such TFs operate, but also into abnormal transcriptional states leading to disease.

## INTRODUCTION

The regulation of gene transcription is a fundamental process whereby cells respond to a multitude of cues that regulate their development and orchestrate specific responses to external stimuli. Transcriptional outputs result from integrating the information encoded by several regulatory signals, including transcription factors (TFs), epigenetics and global chromatin structure inside the nucleus at specific genomic loci (1). Of these signals, TFs are the best understood, as they specifically bind to short DNA sequences, either in basal promoters (where the general transcriptional machinery assembles), or in distal elements, which are responsible for the temporal and tissue-specific expression of genes. ChIP-chip, and, more recently, ChIP-seq technologies, allow the determination of the genome-wide binding sites of specific TFs *in vivo*, and as such have become central tools in the study of transcriptional regulation. In an early example of ChIP-seq done for the TF Tal1 (Scl), an essential regulator of hematopoietic stem cells (HSCs), we found that Tal1 regulates other TFs indispensable for HSC identity and function (2). The computational analysis of the Tal1 binding sites implicated another 10 TFs, which were subsequently validated by ChIP-seq to find that Tal1-bound sites are co-occupied with other TFs, including Lyl1, Gata2, Runx1, Erg, Fli1 and Lmo2 (3). Interestingly, the lack of Runx1 binding sites in a large set of co-occupied genomic sites led to the identification of previously unreported protein–protein interactions (PPIs) between Runx1 and Gata2, Erg and Tal1 [reviewed in (4)]. This shows that combinatorial TF binding is required for driving hematopoiesis, and also that specific PPIs are essential for the assembly of TF complexes. Cooperative interactions between TFs that constitute transcriptional regulatory modules (TRMs) have also been identified in embryonic stem cells (ESCs) (5) plus a number of other cell types through the ENCODE project. In the latter, the conclusions from the analysis of ChIP-seq profiles for 109 TFs in various cell types further emphasized the notion that TFs form TRMs that regulate the temporal and tissue-specific expression of genes (6). TRMs generally extend over short DNA regions and consist of several distinct and interacting TFs that act in concert to perform cell type-specific functions (3,5,7). It has been recognized that a major difficulty in predicting TRMs comes from a lack of understanding of the direct and indirect interactions among TFs and co-factors (8). This is a key aspect, as all proteins need to physically interact with other proteins to perform their functions. Most proteins oligomerize into homomers, and these homomers typically assemble into higher-order structures in specific cellular contexts (9).

The systematic identification of TRMs by ChIP-seq is not presently viable even in those cases where the genome-wide binding sites of a key regulatory TF are known. This is because in the absence of additional information (such as specific TFs previously known to be essential for a particular cell type), we cannot shortlist *a priori* the co-factors whose co-localization should be assessed by ChIP-seq in a specific cell type and context. To fill this gap, here we present a computational framework (rTRM) for the reconstruction of TRMs that exploits information on known PPIs. Given a set of experimentally determined genomic coordinates (e.g. from the ChIP-seq peaks of a target TF), we combine TF motif analysis on these genomic coordinates and cell type-specific gene expression to identify a list of candidate co-factors, and then use rTRM to predict TRMs from PPI networks. We applied rTRM to reconstruct the TRMs characteristic of several embryonic and differentiated cell types, including ESCs, HSCs, neural progenitor cells (NPCs), trophoblast stem cells (TSCs) and several types of differentiated T cells. The prediction of TRMs in ESCs and HSCs in particular yielded dozens of co-factors of which ∼75% have been independently reported to regulate the transcriptional programs of these two cell types. The ability of rTRM to identify key regulatory proteins in ESCs and HSCs (two distinct albeit well-characterized systems) with a high degree of precision suggests that it can be applied to reconstruct TRMs in other cell types and transcriptional contexts.

## MATERIALS AND METHODS

### Overview of rTRM

**Figure 1.**
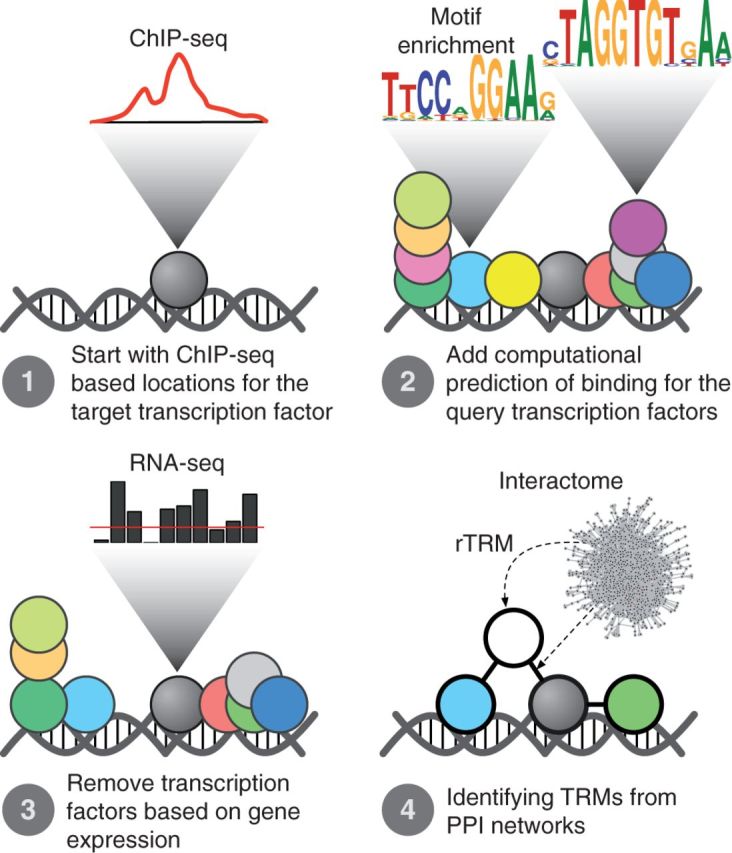
Overview of the rTRM method. Here we illustrate the reconstruction of a TRM around a specific TF whose genomic binding sites have been determined experimentally by ChIP-seq (Step 1). In Step 2, the genomic regions are scanned for enriched motifs against a background. Next, these enriched motifs are mapped to their corresponding TFs while filtering out those genes not expressed in the cellular type under consideration (Step 3). The remaining TFs that putatively bind the set of enriched motifs are mapped onto the species’ PPI network, followed by the application of our own module finding algorithm to identify the TRM.

The goal of the rTRM method is to reconstruct TRMs from a list of candidate TFs using PPI information. To determine the list of candidate TFs, we integrate experimental evidence of TF binding with TF binding site predictions and cell type-specific gene expression data. Briefly, a set of experimentally determined genomic regions are scanned for enriched TF binding motifs, which are subsequently mapped to their corresponding genes. Next, this list of TF genes is filtered to remove non-expressed candidates. Finally, rTRM maps the list of candidate TFs onto the organism’s PPI network, and our own module-finding algorithm is applied to reconstruct a TRM (Figure 1; Supplementary Figure S1 provides a detailed explanation of the computational workflow).

### Compilation of a TF position weight matrix library and mapping to genes and orthologs

Position weight matrices (PWMs) representing the binding specificities of a large number of TFs have been made available in recent years using a diversity of experimental approaches. We compiled a library of 1298 PWMs by integrating the vertebrate entries from the JASPAR database (2010 release, including 130 PWMs from human, mouse, rat, chicken and *Xenopus*) (10), the protein-binding microarray UniPROBE database (299 matrices of human and mouse origin) (11) and the recently published high-throughput SELEX (HT-SELEX) datasets (including 869 PWMs of human and mouse origin) (12,13) (Supplementary Table S1). All 1298 PWMs were uniformly mapped to Entrez Gene identifiers using the original annotations provided in the respective databases (comprising a mixture of Entrez Gene, Uniprot and other protein database identifiers). Finally, all Entrez Gene identifiers were mapped to orthologous genes across species with the Biomart tool as implemented in the biomaRt Bioconductor package (14). The library of 1298 PWMs maps to 548 human TF genes, representing 35–39% of the entire human TF complement as defined by Wingender *et al*. [1558 TF genes, (15)] or by Vaquerizas *et al*. [1391 TF genes (16)].

The TFs mapped onto the PWM library were annotated using the structural classification scheme defined in the TFClass database (15), where human TFs are divided into 9 superclasses, 40 classes and 111 families. Further classification levels include the subclass (optional), gene and ‘Factor species’ (for protein isoforms). Briefly, the OBO-formatted ontology file containing the structural classification of human TFs was downloaded and parsed. ‘Factor species’ entries were removed, as the gene level is the most basic unit used in our analysis pipeline. The mouse TFs were assigned to the specific structural classes of the TFClass database by means of the Biomart-derived table of mouse–human orthology relationships. A concentric graph representing the relationship between the different levels of the TFClass hierarchy and the mapping is shown in Supplementary Figure S2. Finally, all datasets including PWMs, orthology mapping and TFClass classification were organized into tables and stored in an SQLite database. The rTRM package provides an API for easy data query.

### ChIP-seq datasets and *de novo* motif enrichment analysis

rTRM was used to reconstruct the TRMs of mouse ESCs, HSCs, NPCs, TSCs and of several differentiated T cells. As the starting point in the analysis pipeline is a set of experimentally determined genomic coordinates, we relied on publicly available TF-specific ChIP-seq datasets. For ESCs, two distinct datasets were used: (i) the ChIP-seq libraries from Chen *et al*. (5) included the following 13 factors: Ctcf, E2f1, Esrrb, Klf4, Myc, Mycn, Nanog, Pou5f1 (Oct4), Smad1, Sox2, Stat3, Tfcp211 and Zfx; (ii) the ESC ChIP-seq libraries from Lodato *et al*. (17) included Sox2 and Pou5f1 (Oct4), as well as ChIP-seq data for Sox2 and Brn2 (Pou3f2) performed in NPCs. For HSCs, the 10 TFs profiled by Wilson *et al*. (3) included Erg, Fli1, Gata2, Gfi1b, Lmo2, Lyl1, Meis1, Sfpi1 (Pu.1), Runx1 and Tal1 (Scl). For TSCs, binding information for Cdx2, Elf5 and Eomes was obtained from Chuong *et al*. (18). For studying the distinct populations of CD4^+^ T cells, the genome-wide Gata3 binding patterns described in Th1, Th2, Th17 and iTreg cells were obtained from Wei *et al*. (19). For all the above TF-specific ChIP-seq libraries, the summits of all peaks were taken as originally reported and extended 200 bp on either side to generate a collection of 400 bp regions.

*De novo* motif enrichment analysis was performed with HOMER (7) for each ChIP-seq library using default parameters on all 400-bp TF-binding regions. HOMER uses sets of background sequences for enrichment analysis by randomly selecting genomic sequences that possess features similar to the sequences tested for enrichment, including GC content and length. A consequence of this is that every run of HOMER typically produces slightly different enrichment results depending on the set of background sequences. To overcome this bias we performed 10 replicate HOMER runs for each TF and selected only those motifs that were enriched (*q* < 0.05) in at least 80% of the replicates. The final list of enriched motifs was matched against our PWM library using Tomtom (*q < 0.0*5) (20).

### Gene expression datasets

Microarray expression data were obtained from the NCBI Gene Expression Omnibus for ESCs [GSE27708 (21) and GSE38850 (17)], adult bone marrow HSCs [GSE37000 (22)] and NPCs [GSE38850 (17)]. The raw expression data (Affymetrix MOE430 2.0 platform) were processed using the custom chip description file (CDF) files from the BrainArray project (23), and robust multi-array average (RMA) was used for background correction, normalization and summarization (24). For each dataset, we plotted the distribution of expression values to determine the specific cutoffs that allow distinguishing expressed from non-expressed genes. The choice of cutoff values was based on the distribution of background at low intensities (Gaussian distribution) and the specific expression values of key TFs with mutually exclusive biological functions (Supplementary Figure S3). For instance, Pou5f1 (Oct4) and Sox2 are expressed in ESCs but not HSCs, and these were used to determine the expression cutoff in HSCs (log_2_ intensity cutoff value = 5.5). On the other hand, Sfpi1 (Pu.1) and Gata2 are expressed in HSCs but not in ESCs, and so these were used to determine the expression cutoff in ESCs (log_2_ intensity cutoff value = 7.0). For TSCs and the CD4^+^ T cell subsets, paired RNA-seq datasets were obtained from the original publications (GSE42207 and GSE20898, respectively). Raw counts were converted to counts per million (CPM) and a CPM > 1 cutoff was used to determine the list of expressed genes.

### PPI datasets

Mouse PPI data were obtained from the BioGRID database (version 3.2.98, release of March 2013) (25). We removed the edges corresponding to non-physical (genetic) interactions, as well as the ubiquitination and sumoylation moieties Ubc, Sumo1, Sumo2 and Sumo3. The final working PPI network was simplified to maintain only a single edge per pair of proteins, and the original number of edges was recorded as an edge attribute to determine the significance of the interaction. For the cell type-specific reconstruction of TRMs, the whole PPI network was further simplified by removing those proteins (nodes) whose corresponding genes were not expressed in the cell type under study.

### Reconstruction and comparison of TRMs

A local search algorithm was developed to reconstruct TRMs. Our algorithm works by finding proteins in PPI networks separated by a maximum network distance to a target protein, as detailed in Supplementary Figure S4. The target protein is defined as the TF used in the immunoprecipitation step of the ChIP-seq experiment. Briefly, in the first step, the proteins directly connected to the target TF (closest neighbors), as well as the enriched TFs, are retrieved. This is followed by the extraction of the subnetwork of shared nodes, including all neighbors. Finally, those nodes in the subnetwork that are separated from the target TF by more than a maximum set distance are removed. To account for all the equivalent paths connecting any two nodes in the network, all the shortest paths were computed. The shortest path function returns all the nodes connecting any two nodes in the network, including the target nodes themselves. This means that for directly connected nodes, the shortest path results in two nodes, which corresponds to a distance of 1 in the network (as measured by the number of edges connecting two nodes). By default, a maximum of three nodes in the shortest path (distance of 2) was used in this study to allow for the presence of one bridge protein. In rTRM, this parameter can be adjusted by specifying the maximum number of bridge proteins (by default maximum bridge = 1).

The similarity between any two TRMs was determined by calculating the number of common edges. The advantage of using common edges over common nodes to assess the similarity between two networks is illustrated in Supplementary Figure S5. Venn diagrams were used throughout to report the degree of similarity of any two networks. However, to find groups of TRMs with similar structures when comparing multiple networks, a matrix was computed to record the pairwise network Jaccard index of the common edges (see Supplementary Methods), followed by hierarchical clustering using *Pearson* distance and complete linkage (*Pearson* distance *d = 1 – r*, where *r* represents the *Pearson* correlation coefficient).

### Gene Ontology and phenotype analysis

Gene Ontology enrichment analysis was performed for the Biological Process category using the GOstats Bioconductor package (26). A conditional test by Alexa *et al*. that considers the dependency structure of GO terms was performed to avoid the enrichment of duplicated or dependent terms (27). Results were deemed significant at *P < 0.05*. Mouse genetic deletion phenotypes were obtained from the Mouse Genome Database (28), and the enrichment of phenotypes associated with the TRMs was determined with a hypergeometric test using the *R* function *phyper*.

### Availability of rTRM

The rTRM method is licensed under GPL-3 terms and freely available as a package in the *R* programming language (29). The rTRM version used to produce the results presented in this manuscript (rTRM v. 0.9.4) is available at https://sourceforge.net/projects/rtrm. rTRM is also available in Bioconductor (currently in the development version) and future updates to the package will be made available through the Bioconductor Web site (http://www.bioconductor.org). Specific details on rTRM installation and software dependencies are available in the Supplementary Material.

## RESULTS

### A novel method for the reconstruction of TRMs

We have developed a novel method (rTRM) for the reconstruction of TRMs from PPI networks that takes advantage of experimentally determined genomic binding sites for a specific TF (the ‘target TF’). We integrate motif enrichment analysis on the experimentally characterized binding regions and gene expression data to determine a set of candidate TFs. The mapping of TF gene identifiers to a common organism (ortholog mapping) allows TFs to be filtered by expression, and to be mapped onto PPI networks. Then, we use a specific module-finding algorithm to identify TRMs from the list of candidate TFs and the PPI network (Figure 1).

### TRMs controlling mouse ESC identity

As proof of principle, we tested the ability of rTRM to reconstruct the TRM of mouse ESCs. The TFs essential for ESC identity have been extensively characterized (5,30–32), and ChIP-seq libraries are available for 13 distinct TFs [the ‘target TFs’: Ctcf, E2f1, Esrrb, Klf4, Myc, Mycn, Nanog, Pou5f1, Smad1, Sox2, Stat3, Tfcp2l1 and Zfx (5)], all of which regulate distinct aspects of ESC identity. For instance, Pou5f1 (Oct4), Sox2 and Klf4/Esrrb are sufficient to induce the reprogramming of fibroblasts into pluripotent stem cells (33,34), whereas Smad1 and Stat3 are involved in the regulation of the signaling pathways mediated by BMP and LIF, respectively [both pathways being essential for ESC self-renewal (35)]. Moreover, Esrrb and Zfx are involved in the maintenance of ESCs (36,37); E2f1 plays a major role in cell cycle regulation (38); and Ctcf is known to regulate the 3D architecture of chromatin (39).

The peaks reported for all target TFs were analyzed with HOMER to identify enriched DNA-binding motifs. The *de novo* enriched motifs were subsequently matched against our library of PWMs, and then mapped onto mouse Entrez Gene identifiers. TF genes were filtered out by expression using the ESC microarray expression data published by Ho *et al*. (21), and then mapped onto the mouse PPI network to compute TRMs. The only exception was Zfx, which was not found in the PPI network and therefore a TRM could not be built. Moreover, because the rTRM analysis on Ctcf binding sites did not return a TRM (even when applying a maximum distance of 2), we ended up with 11 distinct TF-specific TRMs (Supplementary Figure S6). Supplementary Tables S2 and S3 include specific details on the enriched motifs and genes constituting the final TF-specific TRMs. The predicted TRMs (Supplementary Figure S6) displayed a diversity of TF structural classes, including homeodomain, nuclear receptor, basic helix-loop-helix (bHLH), Rel (RHR), Stat and basic leucine zipper. In general, the TRMs harbor a large number of highly connected TFs (especially those of Stat3 and Mycn). Importantly, all the 11 reconstructed TRMs recovered a high percentage of the target TFs. For instance, the Klf4, Pou5f1, Smad1, Stat3 and Tfcp2l1 TRMs included 6 of the 11 target TFs (54%), whereas the E2f1, Myc, Nanog and Sox2 TRMs included 5 of 11 target TFs (45%). The TRMs recovering the smallest number of target TFs were those of Esrrb (4/11 or 36%) and Myc (3/11 or 27%). This result shows that rTRM is capable of identifying TFs critical for ESC biology starting from a single ChIP-seq library of a target TF.

To assess the reproducibility of the TRMs predicted in ESCs, we predicted TRMs for Sox2 and Pou5f1 (Oct4) also done in ESCs but in a different laboratory. Besides the ChIP-seq studies for Sox2 and Oct4, Lodato *et al*. (17) also produced paired RNA-seq expression data, which were used to determine the expression level cutoff (CPM > 1 for all samples). Supplementary Figure S7A shows a comparison of the Sox2 TRMs built from the data of Chen *et al*. and Lodato *et al*.: the Lodato Sox2 TRM is larger than the Chen TRM, but most of the proteins and interactions are found in both networks. Moreover, ∼75% of the nodes (proteins) and edges (interactions) in the Chen TRM are found in the Lodato TRM (Supplementary Figure S7B). The larger number of proteins and interactions found in the Lodato TRM can be explained by the greater sequencing depth of the Lodato ChIP-seq experiment, which resulted in nearly three times more peaks than the Chen dataset (Supplementary Figure S7B). A consequence of this is the identification of a larger number of enriched TFs (Supplementary Figure S7B). For Pou5f1 (Oct4), we found almost identical results (Supplementary Figure S7C), suggesting that rTRM is capable of predicting comparable TRMs for the same TF profiled by different laboratories.

**Figure 2.**
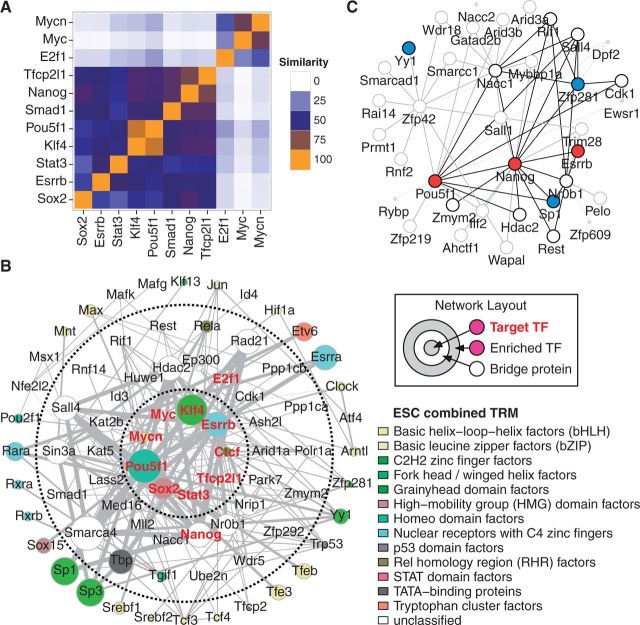
TRMs predicted in ESCs. (**A**) Heatmap displaying the degrees of similarity among the 11 distinct TRMs as measured by the Jaccard index. (**B**) The combined TRM identified in ESCs as an amalgamation of the 11 distinct TRMs for each specific target TF. The size of the nodes and edges is proportional to their presence in the individual TRMs. (**C**) Comparison between the ESC-combined TRM and the ESC PPI network. Black lines represent proteins and interactions identified by rTRM.

The relevance of the 11 reconstructed TRMs to ESC biology was determined using a variety of strategies. First, we calculated the similarity among the 11 TRMs as the *Jaccard* index of shared interactions (edges) (Figure 2A; Supplementary Methods and Supplementary Figure S5) and identified two distinct clusters: one containing Mycn, Myc and E2f1, and a second cluster including the other eight TRMs. Within the larger cluster, the Pou5f1 and Klf4 TRMs share the highest degree of similarity (85%), followed by Smad1, Nanog and Tcfcp2l1 (68%), and Esrrb and Stat3 (58%). The TRMs constituting the first cluster (Mycn, Myc and E2f1) displayed a smaller degree of similarity (42%). These results support the existence of two independent TRMs in ESCs: a larger one composed by Esrrb, Klf4, Nanog, Pou5f1, Sox2, Stat3, Smad1 and Tfcp2l1, and a smaller one composed by E2f1, Myc and Mycn. This observation is in agreement with the cluster membership previously identified in the analysis of the genome-wide binding site profiles where ESC TFs were found to be wired into two separate clusters: one containing Nanog, Oct4, Sox2, Smad1 and Stat3, and a second cluster containing E2f1, Myc, Mycn and Zfx (5). Second, the functional relevance of the Esrrb and Sox2 TRMs was determined by mapping the constituent genes to expression data obtained from Esrrb, Sox2 and Esrrb/Sox2 double knockdowns (37). We found that most of the genes were downregulated in the knockdowns compared with the GFP control, suggesting that these regulatory networks are functional and interdependent, as they support each other’s gene expression patterns (Supplementary Figure S8). Next, we compared the predicted TRMs with the ESC PPI network published by Wang *et al*. (31), who combined affinity purification with mass-spectrometry to determine the interacting partners of Nanog and other proteins crucial for ESC biology (Pou5f1/Oct4, Nr0b1/Dax1, Nacc1/Nac1, Zfp281 and Zfp42/Rex1). To do this, the 11 distinct TRMs were combined into a single unified TRM (the ‘ESC-combined TRM’) consisting of 77 proteins and 170 interactions (Figure 2B; Supplementary Table S4). The ESC-combined TRM was plotted using the same concentric layout as before. To highlight the importance of each of the identified proteins/interactions in the ESC transcriptional regulatory network, the sizes of the nodes and the widths of the edges were scaled to represent the abundance of the individual proteins and interactions across the 11 distinct TF-specific TRMs. For instance, Esrrb, Klf4, Nanog, Pou5f1, Smarca4, Sox2 and Sp1 constitute the larger nodes in the ESC-combined TRM because they are present in the majority of the 11 TRMs. However, the Myc and Mycn nodes are relatively small because they could only be found in the E2f1, Myc and Mycn TRMs. The PPI network described by Wang *et al*. contains 37 proteins and 69 interactions, and the analysis of the overlap with the ESC-combined TRM resulted in 13 proteins and 27 interactions in common (Figure 2C). These common proteins included the target TFs Pou5f1, Nanog and Esrrb (red nodes in Figure 2C), and the following enriched TFs: Sp1, Zfp281 and Yy1 (light blue nodes in Figure 2C). This observation adds further support to their role as co-regulators in the ESC transcriptional network. Sp1 (or, in its absence, Sp3) has been reported to bind to the promoter of Pou5f1 (Oct4) to regulate its expression (40), and Zfp281 is known to directly activate Nanog expression as well as being required to maintain ESCs (41). Interestingly, Cdk1, Hdac2, Nacc1, Nr0b1, Rest, Rif1, Sall4 and Zmym2, which either lack a corresponding PWM or are not predicted to bind DNA directly, were also found both in the ESC-combined TRM and the PPI network by Wang *et al*. (31). This highlights an important characteristic of the TRMs identified by rTRM, which is that important ESC transcriptional regulators were reported as bridge proteins. For instance, among the bridge proteins included in the ESC-combined TRM (Figure 2B), Nr0b1 is essential for maintaining the ESC phenotype (42), as well as an important determinant for specifying ESCs over the closely related epiblast stem cells (37). Sall4 is also essential for maintaining pluripotency in ESCs by regulating the expression of Pou5f1 (Oct4) (43), and Arid1a is a member of the ESC SWI/SNF chromatin remodeling complex (44). Finally, Sin3a is known to interact with Nanog (45) and is also essential for ESCs (46). Overall, these results demonstrate that rTRM recovers known regulatory complexes and that the predicted TRMs are especially enriched in TFs and other regulatory proteins important for determining the ESC phenotype.

Gene functional enrichment analysis using the Gene Ontology (GO) Biological Process category on both the 11 TRMs and the ESC-combined TRM identified specific terms common to all TRMs and related to ESC biology (Supplementary Figure S9). The genes Esrrb, Klf4, Msx1, Nanog, Pou5f1, Rif1, Sall4, Smarca4, Sox2 and Stat3 were all associated with ‘stem cell differentiation’ (*P = 2.1e-13 ∼ 5.5e-3*), whereas Esrrb, Klf4, Rif1, Sall4, Smarca4 and Stat3 were associated with ‘stem cell maintenance’ (*P = 2.7e-8 ∼ 2.9e-2*). Enrichment analysis using the genetic deletion phenotypes from the Mouse Genome Informatics database showed that ∼17% (13/77) of the nodes in the ESC-combined TRM were associated with ‘complete embryonic lethality between implantation and somite formation’ (*P ∼ *0) and/or ‘partial embryonic lethality between implantation and somite formation’ (*P = 1.5e-5*) (Table 1), thus providing further support for the biological relevance of the nodes in the ESC-combined TRM.

**Table 1.** List of embryonic lethal proteins identified in the ESC-combined TRM

Entrez gene	Symbol	Embryonic lethal
**93760**	Arid1a	Complete
**15251**	Hif1a	Partial
**18519**	Kat2b	Complete
**17187**	Max	Complete
**71950**	**Nanog**	Complete
**18999**	**Pou5f1**	Complete
**99377**	Sall4	Complete
**20466**	Sin3a	Complete
**20674**	**Sox2**	Complete
**20848**	**Stat3**	Complete
**22059**	Trp53	Complete
**93765**	Ube2n	Complete
**226442**	Zfp281	Complete

Genes were selected based on embryonic lethal phenotype obtained from the Mouse Genome Database. Complete (MP:0011096) indicates ‘complete embryonic lethality between implantation and somite formation’, whereas partial (MP:0011106) indicates ‘partial embryonic lethality between implantation and somite formation’. Target TFs included in the published ChIP-seq experiments are highlighted in bold.

Finally, we investigated the published literature for independent evidence of the 77 proteins of the ESC-combined TRM as having a role in ESC biology. We found that ∼72% of the genes in the ESC-combined TRM had been independently reported to play fundamental roles in the biology of ESCs (Supplementary Table S4).

### Comparison between embryonic and HSC TRMs

**Figure 3.**
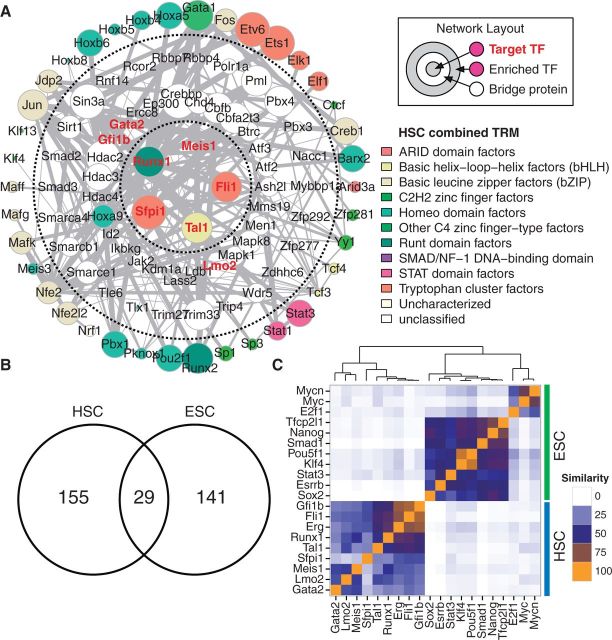
Comparison between the combined ESC and HSC TRMs. (**A**) Combined TRM for HSCs. The size of the nodes and edges is proportional to their presence in the individual TRMs. (**B**) Number of edges shared between the ESC and HSC-combined TRMs. (**C**) Heatmap displaying the Jaccard index of edges shared between the individual ESC and HSC TRMs. Groups of TRMs with similar network structures were identified using hierarchical clustering.

Hematopoiesis is possibly the best-understood model of adult stem cell development and differentiation (47–51), where a large number of essential TFs have been identified largely from specific mutations that lead to hematopoietic malignancies (4,52,53). Wilson *et al*. (3) previously reported the genome-wide binding locations of 10 key HSC TFs, including Erg, Fli1, Gata2, Gfi1b, Lmo2, Lyl1, Meis1, Sfpi1, Runx1 and Tal1/Scl. Following the same workflow described above for ESCs, we generated 9 independent TRMs for each TF and then produced a combined TRM for HSCs (Figure 3A and Supplementary Figure S10). Lyl1 was not present in the PPI network and hence a corresponding TRM could not be reconstructed. Visual inspection of the 9 TRMs highlighted a preference for homeodomain (13 proteins), bHLH (9 proteins), C2H2 zinc finger (7) and tryptophan cluster factor members (ETS family TFs, some of which were already known to be essential for HSCs, such as Sfpi1 and Fli1). Supplementary Table S2 gives details on the numbers of motifs and genes at each stage in the analysis pipeline, whereas Supplementary Table S3 lists the proteins included in each individual TRM together with their classification and role in the network. Supplementary Table S4 provides a list of the proteins included in the HSC-combined TRM.

GO enrichment analysis for the Biological Process category returned specific terms associated both with stem cell and hematopoietic cell biology, including ‘germ-line stem cell maintenance’, ‘regulation of stem cell maintenance’, ‘stem cell maintenance’ and ‘stem cell differentiation’. In addition, the terms ‘hemopoietic stem cell differentiation’ and ‘hemopoietic stem cell proliferation’ were enriched in 8 of 10 modules (*P = 0.01 ∼ 0.04*) (Supplementary Figure S11). Moreover, examination of the genes in the HSC-combined TRM identified 22 of 96 (23%) as leading to impaired hematopoiesis on genetic deletion, and the following terms as being significantly enriched: ‘impaired hematopoiesis’ (*P ∼ 0*), ‘abnormal hematopoiesis’ (*P = 2.9e-11*), ‘abnormal blood cell morphology/development’ (*P ∼ 0*) and ‘abnormal lymphopoiesis’ (*P = 8.9e-6*) (Table 2). Collectively, these results suggest an enrichment of hematopoietic factors in the combined TRM predicted for HSCs. Finally, an in-depth investigation of the published literature on the 96 proteins constituting the HSC-combined TRM returned 76% of genes as having independent experimental evidence for specific roles in HSC biology (Supplementary Table S4). For instance, Ldb1 is essential for HSC maintenance in the mouse, where it forms a complex with Lmo2, Tal1 (Scl) and Gata1 (or Gata2) (54). Therefore, besides recovering TFs with known roles in HSCs, the HSC-combined TRM contained additional proteins of specific importance for blood stem cells.

**Table 2.** List of proteins identified in the HSC-combined TRM with abnormal/impaired hematopoiesis

Entrez gene	Symbol	Impaired hematopoiesis
**12400**	Cbfb	Hematopoiesis
**12914**	Crebbp	Hematopoiesis
**23871**	Ets1	Lymphopoiesis
**14011**	Etv6	Hematopoiesis/lymphopoiesis
**14247**	**Fli1**	Hematopoiesis
**14281**	Fos	Lymphopoiesis
**14460**	Gata1	Hematopoiesis
**14461**	**Gata2**	Hematopoiesis/morphology
**14582**	**Gfi1b**	Morphology
**15412**	Hoxb4	Hematopoiesis
**15414**	Hoxb6	Hematopoiesis
**16452**	Jak2	Hematopoiesis
**16909**	**Lmo2**	Morphology
**17268**	**Meis1**	Hematopoiesis
**18514**	Pbx1	Hematopoiesis
**12394**	**Runx1**	Hematopoiesis
**12393**	Runx2	Hematopoiesis
**20375**	**Sfpi1**	Hematopoiesis/morphology
**20586**	Smarca4	Morphology
**20587**	Smarcb1	Morphology
**21349**	**Tal1/Scl**	Hematopoiesis
**21423**	Tcf3	Lymphopoiesis/morphology

Genes were selected based on altered hematopoietic phenotype, including ‘impaired hematopoiesis’ (MP:0001606), ‘abnormal hematopoiesis’ (MP:0002123), ‘abnormal blood cell morphology/development’ (MP:0002429) and ‘abnormal lymphopoiesis’ (MP:0002401). ‘Hematopoiesis’ below stands for ‘impaired hematopoiesis’ and/or ‘abnormal hematopoiesis’, ‘Lymphopoiesis’ stands for ‘abnormal lymphopoiesis’ and ‘Morphology’ stands for ‘abnormal blood cell morphology/development’. Target TFs included in the ChIP-seq experiments are highlighted in bold.

We compared the combined TRMs of ESCs and HSCs (which characterize two distinct types of stem cell, embryonic and mature) and found that both networks share 27 distinct proteins. Comparing the number of edges yielded 29 (16%) interactions in common between the HSC and ESC-combined TRMs, with 141 (82%) edges being unique to ESCs and 155 (84%) edges being specific to HSCs (Figure 3B). An all-against-all pairwise comparison of the percentage of shared edges among the 20 TRMs identified in ESCs and HSCs (Supplementary Figures S6 and S10), followed by hierarchical clustering, identified two clearly distinct clusters: one containing all the TRMs of ESCs, and a second cluster harboring all the TRMs of HSCs (Figure 3C). This indicates that although some of the proteins and edges identified in the TRMs are common to both ESCs and HSCs, the regulatory pathways connecting the TFs in the two types of stem cell are actually different. Therefore, rTRM is capable of identifying TRMs with clearly distinct and cell type-specific features in the two types of stem cells.

### Identification of developmental stage-specific TRMs

Besides identifying highly relevant cell type-specific TRMs in ESCs and HSCs, we also tested the ability of rTRM to reconstruct TRMs in other embryonic/precursor cells as well as in differentiated CD4^+^ T cells. TRMs were identified for Cdx2 in TSCs, for Sox2 in NPCs and for Gata3 in Th1, Th2, Th17 and iTreg cells, with all resulting TRMs shown in Supplementary Figure S12. The Cdx2-based TRM for TSCs included some proteins essential for the determination and function of trophoblast cells. For instance, Runx1 is known to regulate the expression of Ada, a gene expressed in placental trophoblast cells that plays a fundamental role in the developing embryo (55), Smarca4 is important for TSC maintenance (56) and Rnf2 (also known as Ring1b, a polycomb protein) inactivates the X chromosome in developing female embryos (57). The Sox2-based TRM for NPCs is remarkably different from the Sox2 TRM from ESCs: whereas nuclear receptors with C4 zinc fingers were predicted in ESCs (including Esrra and Esrrb), other TF families seem to be specific to the Sox2 TRM of NPCs, including bHLH (Tcf3, Tcf4), Fork head (Hoxa1, Hoxa5), Paired box (Pax6), SAND (Gmeb2), Tryptophan cluster (Etv6) and STAT (Stat3). The distinct TRMs identified around Gata3 in CD4^+^ T cells presented important similarities (which is understandable, as all these cell types are developmentally close), as well as differences. To provide a comprehensive snapshot of all these T cell TRMs, all networks were combined and the nodes colored according to the cell type where they had been predicted (Supplementary Figure S13). Most nodes are common to all cell types (red nodes), whereas others are specific to iTreg cells (blue nodes), suggesting that iTreg cells are the most functionally distinct cell type. Even though all these T cell types have clearly distinct functions, the interpretation of these graphs is problematic, given the cells’ developmental proximity, and probably additional factors such as epigenetic and temporal regulation, should be taken into account in these cases. For instance, Runx1 is a factor common to all T cells, but under specific circumstances, it is known to inhibit the differentiation of naïve CD4^+^ T cells into Th2 cells by repressing Gata3 expression, another common factor (58). Furthermore, the interaction between Runx1, RORgt and Foxp3 regulates the differentiation of the Th17 route (59).

**Figure 4.**
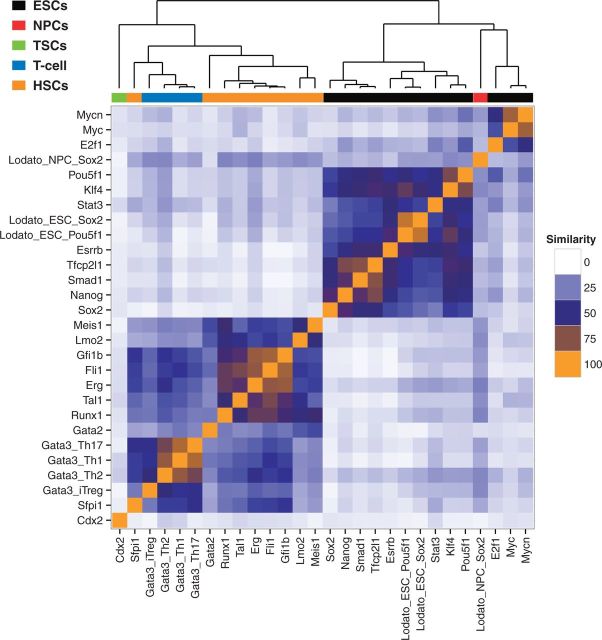
Comparison of all the TRMs identified in this study. Heatmap displaying the Jaccard index of edges shared between the individual TRMs identified in ESCs, HSCs, TSCs, NPC and CD4^+^ T cells. TRMs with similar network structures were identified using hierarchical clustering.

Finally, we compared all the TRMs identified in this study using the *Jaccard* index of the shared edges followed by hierarchical clustering (Figure 4). The TRMs clustered by cell type, clearly separating the ESC, NPC, HSC and TSC TRMs. Interestingly, the HSCs and CD4^+^ T cell TRMs clustered together, with Sfpi1 (Pu.1) displaying similarity to both HSCs and CD4^+^ T cells. This suggests a critical role for Sfpi1 (Pu.1) in different aspects of hematopoietic cell development, which is supported by the high interdependence between Gata3 and Sfpi1 (Pu.1) (60,61).

## DISCUSSION

We have implemented a framework (rTRM) for the systematic identification of TRMs from PPI networks. Using rTRM, we built TRMs specific for the well-studied ESCs and HSCs, which were highly enriched in known regulatory factors in both cell types as ∼75% of the proteins in the corresponding TRMs had been reported to be involved in the function of either ESCs or HSCs. Moreover, the comparison between the ESC-combined TRM and the experimentally determined PPI network by Wang *et al*. (31) demonstrated that rTRM can successfully identify additional proteins with known roles in the regulation of ESCs that cannot be found using genomic information alone, thereby emphasizing the advantage of our approach over methods that ignore PPI information. A significant fraction of the reconstructed TRMs were composed of ‘bridge’ proteins, including TFs like Smad1 and Nanog, which did not have a corresponding binding matrix in our collection of PWMs. However, it is possible that once the information on their binding specificities is included, some of these will become part of the ‘enriched’ set of TFs (e.g. Smad1 and Nanog in ESC). Nevertheless, in some TRMs, specific proteins were predicted for which DNA binding specificity information (PWM) is available, although these PWMs were not found to be enriched, genuinely suggesting a putative role as bridge proteins (e.g. Gata1, Hoxa9). This might be indicative of tethering, an increasingly important regulatory mechanism for some TFs (62). Bridging proteins typically also include chromatin modifying and remodeling enzymes, and signaling molecules, all of which lack the ability to bind specific DNA sequences, and which therefore are impossible to detect using sequence-based approaches only.

The reconstruction of TRMs can yield extremely valuable information when experimental datasets are scarce, or when no regulatory proteins have yet been identified. This is especially true for poorly characterized systems such as the IL10/JAK1/STAT3 pathway that drives the anti-inflammatory response in various immune cells and where few co-factors of STAT3 have been identified (63,64). We recently applied rTRM to predict co-factors that determine the cell type-specific and cell type-independent binding modes of STAT3 in four distinct cell types, and experimentally demonstrated by ChIP-qPCR the role of E2f1 as a co-factor of STAT3 in macrophages (65,66).

In recent years, a number of methods have been developed that integrate experimentally determined binding sites with expression data to identify gene regulatory networks. In 2003, Bar-Joseph *et al*. (67) explored the genome-wide regulatory patterns for 106 yeast TFs using epitope tagging in combination with over 500 expression experiments to identify sets of co-expressed genes that were regulated by specific TFs (67). In 2006, Wu *et al*. (68) followed a similar approach but instead included information from ChIP-chip data, also in yeast. Other available tools combine ChIP-seq data with motif analysis (69), sometimes even including spatial constraints to specifically identify cis-regulatory modules in enhanceosomes (37, 70–71). The general limitation of all these methods, however, is that they ignore PPI information and simply focus on the local DNA sequence to discover gene regulatory units. All proteins require physical interactions with other proteins to perform their functions, and therefore PPIs cannot be neglected if we want to understand complex systems of interacting molecules. TRMs function as compact modules, as the proteins in the complex engage in stronger interactions within the complex than with external molecules (9).

In conclusion, elucidating the genetic targets of TFs is crucial for understanding their downstream biological effects, but an equally important piece of information is the combinatorial interactions of key TFs with specific co-factors that aid in the selection of genomic sites, are involved in the modulation of the local epigenetic environment and also integrate multiple signals. rTRM is a novel method that takes advantage of existing PPI information and which can be systematically applied to the reconstruction of TRMs in distinct cell types and biological contexts. Proteins interact with other proteins to form complexes and perform specific functions, and protein modules are often used in a variety of biological contexts (9). Ergo, a more complete description of experimentally validated PPIs in public databases will undoubtedly increase the predictive power of rTRM. rTRM provides researchers with a powerful tool to dissect the multiple levels of regulation of their TFs of interest. The reconstruction and experimental validation of TRMs will provide fundamental insights not only into physiological TF mechanisms but also into deregulated transcriptional networks that lead to pathological states.

## SUPPLEMENTARY DATA

Supplementary Data are available at NAR Online.

## FUNDING

Japan Society for the Promotion of Science (JSPS) through the WPI-IFReC Research Program and a Kakenhi grant; Kishimoto Foundation; ETHZ-JST Japanese-Swiss Cooperative Program (to D.M.S.); Funding for open access charge: WPI research program.

*Conflict of interest statement*. None declared.

## Supplementary Material

Supplementary Data
