# Supplementary material for: Systematic identification of transcriptional regulatory modules from protein–protein interaction networks
Source: Nucleic Acids Res. 2013 Oct 16;42(1):e6. doi: 10.1093/nar/gkt913 (PMC3874207; doi:10.1093/nar/gkt913)
Supplement: Supplementary Data [file supp_42_1_e6__index.html]

Systematic identification of transcriptional regulatory modules from protein–protein interaction networks — Systematic identification of transcriptional regulatory modules from protein–protein interaction networks — Supplementary Data 

# Systematic identification of transcriptional regulatory modules from protein–protein interaction networks

## Supplementary Data

files

**Files in this Data Supplement:**

- Supplementary Data - pdf file
- Supplementary Data - xls file
- Supplementary Data - xlsx file
- Supplementary Data - xls file
- Supplementary Data - xls file
