# Supplementary material for: Systematic identification of transcriptional regulatory modules from protein–protein interaction networks
Source: Nucleic Acids Res. 2013 Oct 16;42(1):e6. doi: 10.1093/nar/gkt913 (PMC3874207; doi:10.1093/nar/gkt913)

Systematic identification of transcriptional regulatory modules from protein-protein interaction networks

Supplementary Material

Diego Diez[1], Andrew Paul Hutchins[1] and Diego Miranda-Saavedra[1*]

[1]Bioinformatics and Genomics Laboratory, World Premier International (WPI) Immunology Frontier Research Center (IFReC), Osaka University, 3-1 Yamadaoka, Suita 565-0871, Osaka, Japan.

[*]Author for correspondence. E-mail. diego@ifrec.osaka-u.ac.jp; Tel. +81 6 6879 4269.

SUPPLEMENTARY METHODS

Overview

rTRM is a method for predicting transcriptional regulatory modules (TRMs) that combines experimentally determined genomic binding sites for a specific TF (e.g. from ChIP-seq), the computational prediction of enriched TF binding motifs, gene expression and protein-protein interaction (PPI) data. rTRM was designed with ChIP-seq experiments in mind but any other readout providing genomic locations of regulatory regions can be readily adapted (e.g. DNase I footprinting).

Availability

rTRM has been implemented as an R package, is licensed under GPL-3 terms and is freely available for download. The rTRM version used for this manuscript (0.9.4) can be found at https://sourceforge.net/projects/rtrm, including the documentation (rTRM_introduction.pdf; also included in the package). rTRM has also been accepted as a Bioconductor package, and is currently available in the development version of Bioconductor (http://bioconductor.org/packages/devel/bioc/html/rTRM.html) and will be generally available from www.bioconductor.org following the next Bioconductor release (October 2013). For instructions on how to install Bioconductor and companion packages, please check the information at http://bioconductor.org/install/. For instructions on how to install the version available at sourceforge please consult the following section.

Installation

This section details the installation process of rTRM using the packages available at sourceforge. This should only we done by those wishing to reproduce the results presented in the manuscript. For everyone else, please use the package available at Bioconductor, which contains further developments and bug fixes, as well as integration with the Bioconductor infrastructure. For instructions on how to install rTRM from Bioconductor please follow the information at http://bioconductor.org/install/. rTRM runs on the R programming language, which can be installed on Windows and Unix-based machines (including Mac and Linux). Binaries and source code for R can be obtained from www.r-project.org. rTRM has been successfully tested in recent versions of R, including R-2.15.x and R-3.0.1. rTRM dependencies include RSQLite (for managing the SQLite database, which itself depends on the DBI package) and igraph (for graph manipulation).

```
# To install dependencies, from the R prompt (any platform):

> install.packages(c("RSQLite", "igraph"))

# To install rTRM from the command line:

$ R CMD INSTALL rTRM_0.9.4.tar.gz
```

Documentation

rTRM comes with documentation including a reference guide for each command and a vignette with a general introduction to the problem of predicting TRMs, a description of the most common operations as well as some examples. The PDF version of the vignette used in this paper is also available at https://sourceforge.net/projects/rtrm.

```
# To access the vignette from the R prompt:

> browseVignettes(package="rTRM")
```

Protein-protein interaction datasets

rTRM relies on PPI datasets to predict TRMs, and so the package includes interaction data (as an *igraph* object) from the BioGRID database (http://www.thebiogrid.org; version 3.2.98) for human and mouse. The rTRM framework provides a facility to retrieve and process updated datasets directly from the BioGRID website. Please consult the package vignette for specific instructions on how to update the PPI dataset.

Computation of network similarities: the Jaccard index

Similarities between TRMs were computed using the Jaccard index, which measures the proportion of shared elements between two sets (intersection) divided by the total number of elements (union; see eq.). In this manuscript we used the Jaccard index of shared edges (Supplementary Figure 5).

$$J(A,B) = \frac{|A \bigcap B|}{|A \bigcup B|}$$

SUPPLEMENTARY FIGURES

Supplementary Figure 1. Schematic representation of the rTRM framework and workflow. The left panel describes the rTRM database module, which comprises the generation of a library of PWMs with annotations of the PWM gene identifiers and the species of origin, mappings of the original gene to orthologous species (currently human and mouse), and the mapping of TFs to the TFClass scheme. The right panel represents the user pipeline, which starts with the identification of enriched motifs from experimentally determined genomic binding sites (from ChIP-seq data). Enriched motifs are automatically mapped onto specific genes of the organism under study. Expression data is used to filter out non-expressed genes, and the genes still remaining are mapped onto the organism's protein-protein interaction network. Finally a TRM is reconstructed from the target gene's neighbourhood using our own module-finding algorithm (rTRM finder).

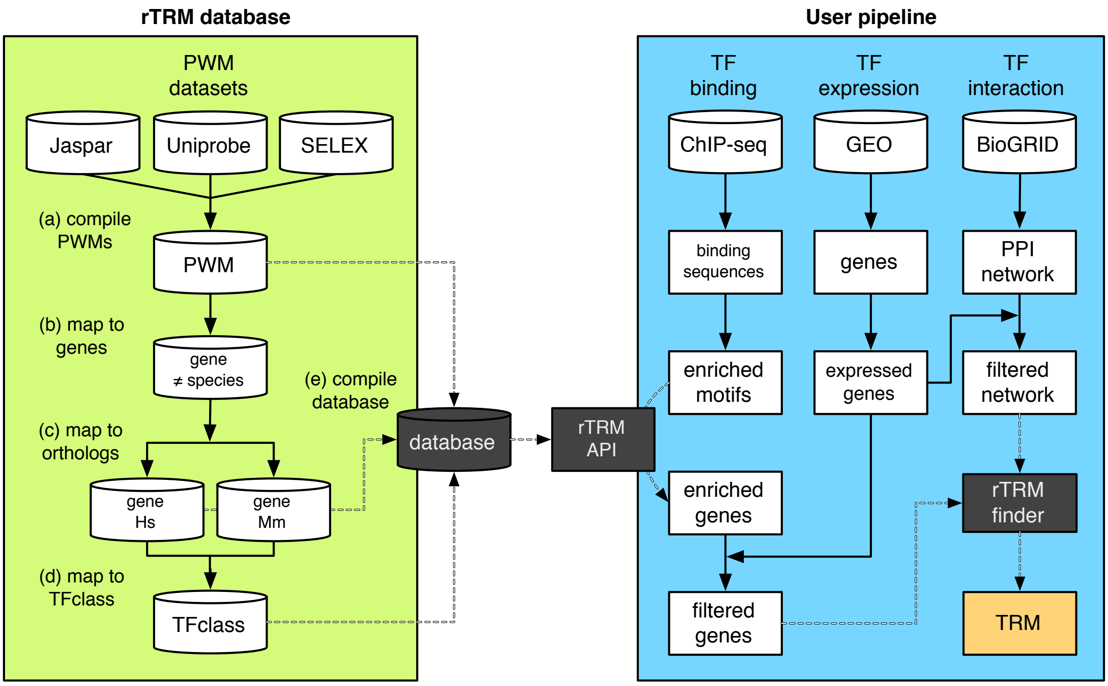

Supplementary Figure 2. TFClass ontology tree. Concentric layout tree showing the mapping of the TFs included in rTRM onto the ontology classification in TFClass.

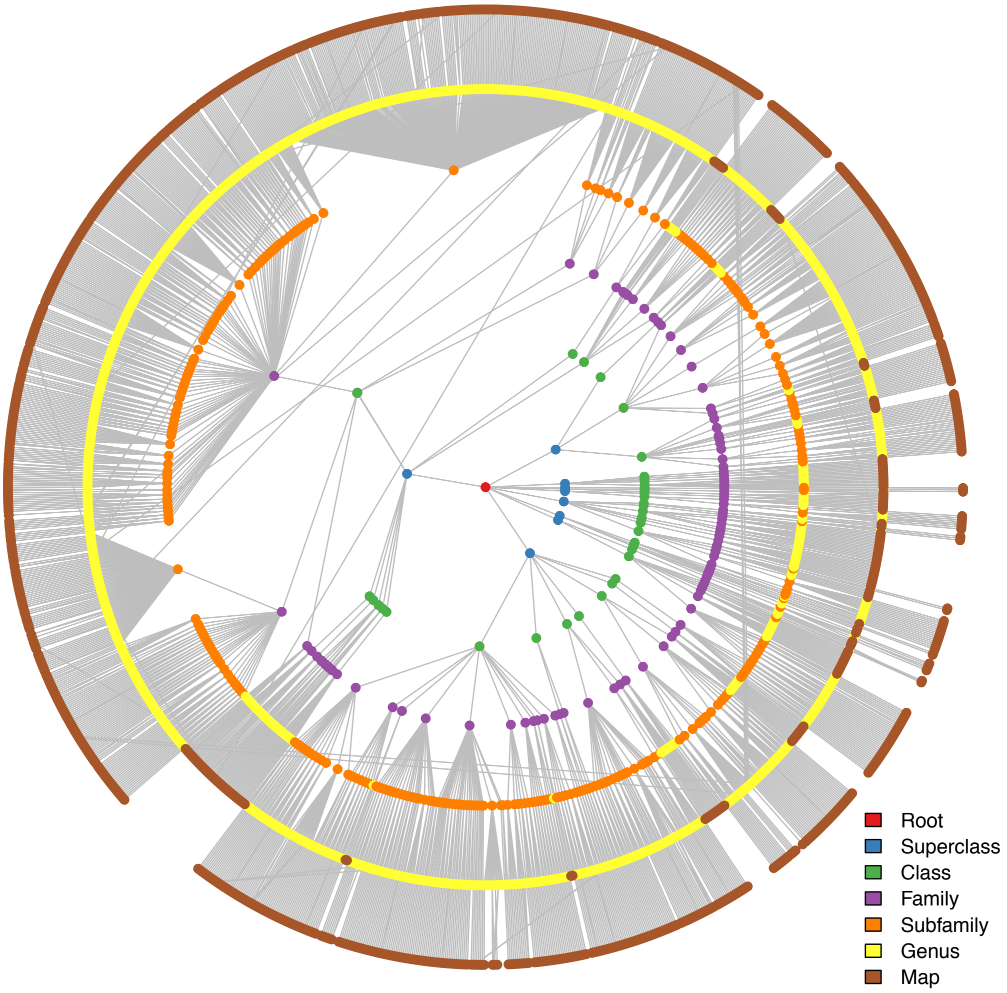

Supplementary Figure 3. Plots showing the distribution of expression values in ESCs and HSCs to determine the expression cutoffs for ESCs (A) and HSCs (B). The distribution of (log_2) intensities was plotted for each individual array. For selected genes, the median intensity across all arrays is indicated. Red lines indicate the filtering cutoff (5.5 for ESCs and 7.0 for HSCs).

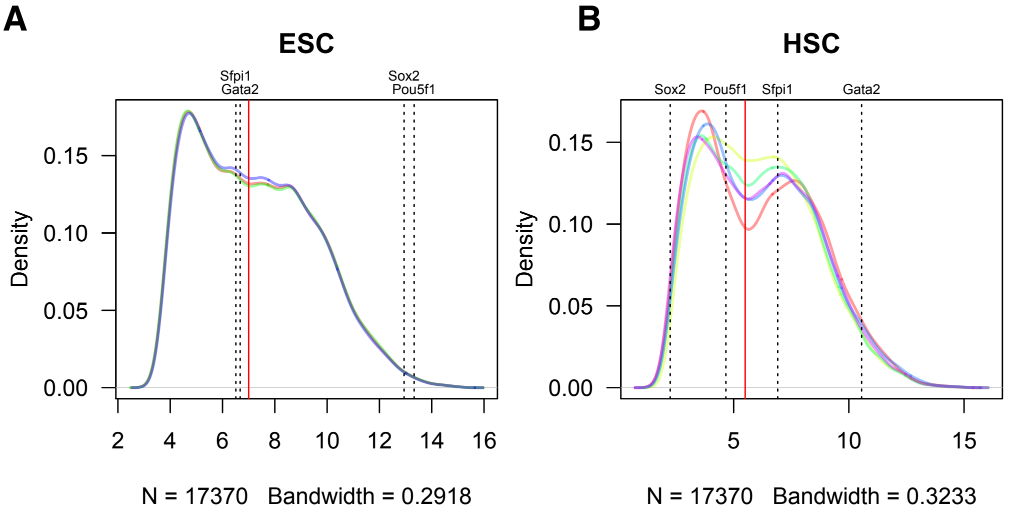

Supplementary Figure 4. Identification of TRMs from PPIs. A neighborhood search algorithm was implemented to identify proteins directly interacting with a target TF or through a bridge protein. First, target TFs are mapped onto the PPI network (1) and then the first neighbours of each TF are identified (2). Next, the common subgraph containing all the interactions for the selected nodes is extracted (3), and finally distance restrictions (maximum separation of one node) are applied and the resulting TRM is returned.

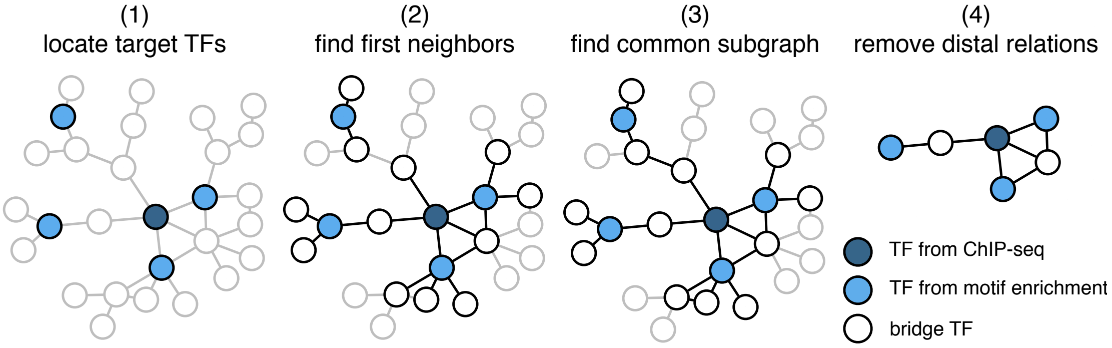

Supplementary Figure 5. Computing network similarity. In order to compare any two networks, two distinct properties may be used: the number of common nodes and the number of common edges. In this figure Networks A and B have five nodes in common (A-E) but which are connected in distinct ways (shown in blue). Network A has one unique node (F; green) and Network B has another unique node (G; red). The similarity based on the number of common nodes is represented in the Venn diagram in the bottom left corner. In contrast, the analysis of the number of common edges between Networks A and B results in two common edges (indicated in blue). Network A has three unique edges (green) whereas Network B has two unique edges (red), as shown in the Venn diagram for edges in the bottom right corner. Based on this, we used the comparison of edges as a more reliable estimation of the degree of similarity between networks.

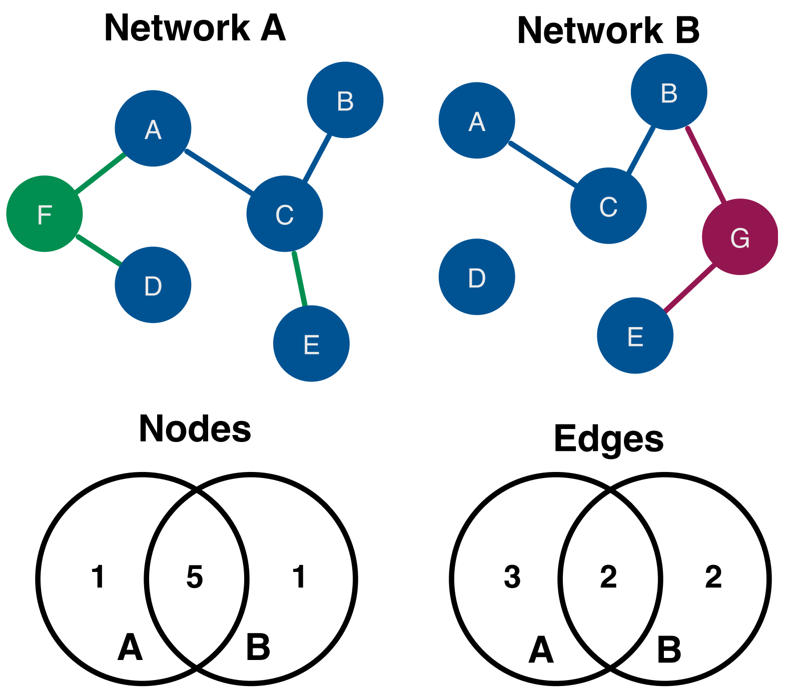

Supplementary Figure 6. Individual TRMs identified in ESCs.

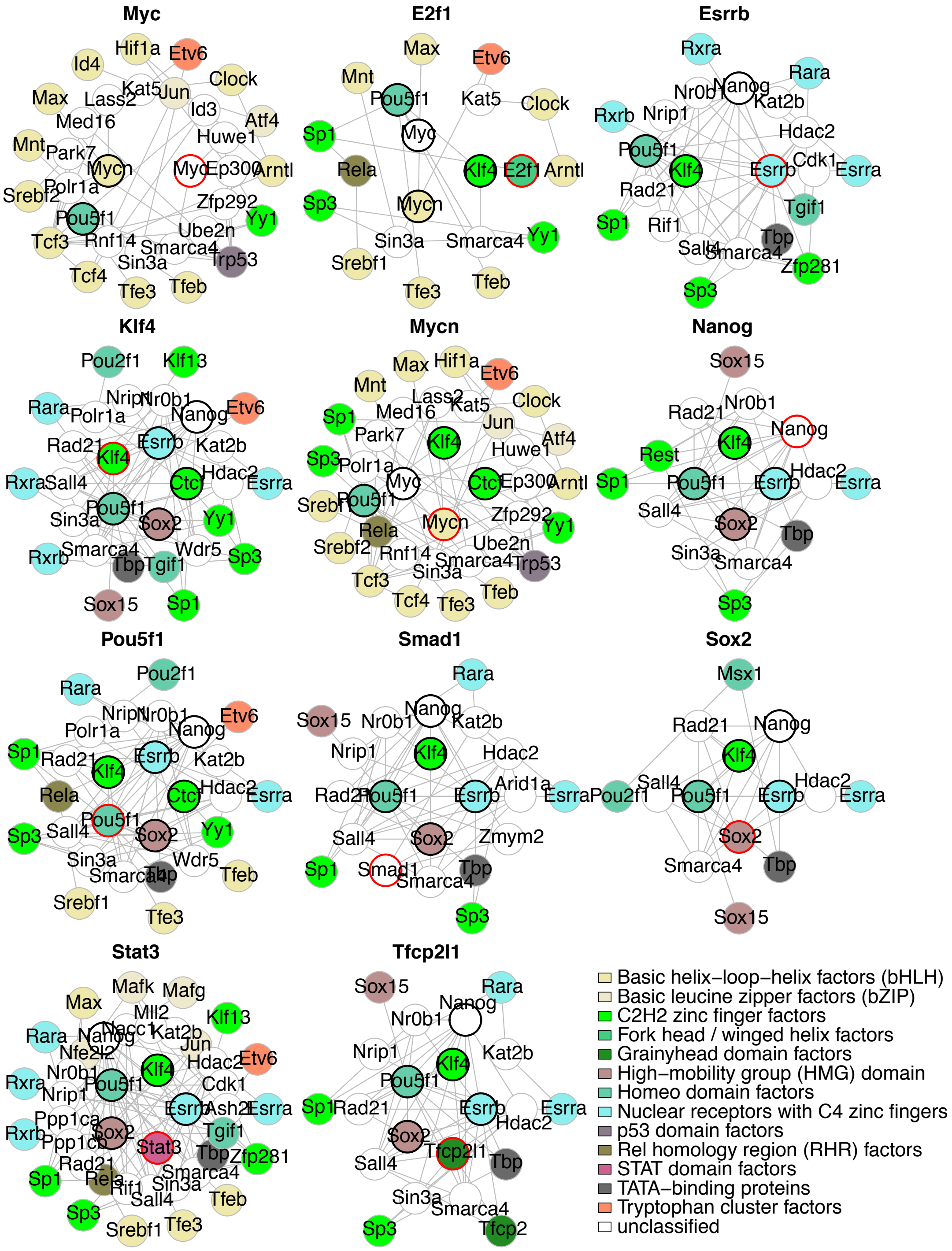

Supplementary Figure 7. Reproducibility of rTRM predicted TRMs in different studies. (A) TRM predicted for Sox2 obtained using information from the Chen and Lodato datasets. (B) Comparison of the number of shared nodes and edges between the Chen and Lodato Sox2 TRMs, referred to the Chen TRM, as well as differences in the number of peaks and enriched motifs. (C) Comparison of the number of shared nodes and edges between the Chen and Lodato Pou5f1 (Oct4) TRMs, referred to the Chen TRM, as well as differences in the number of peaks and enriched motifs.

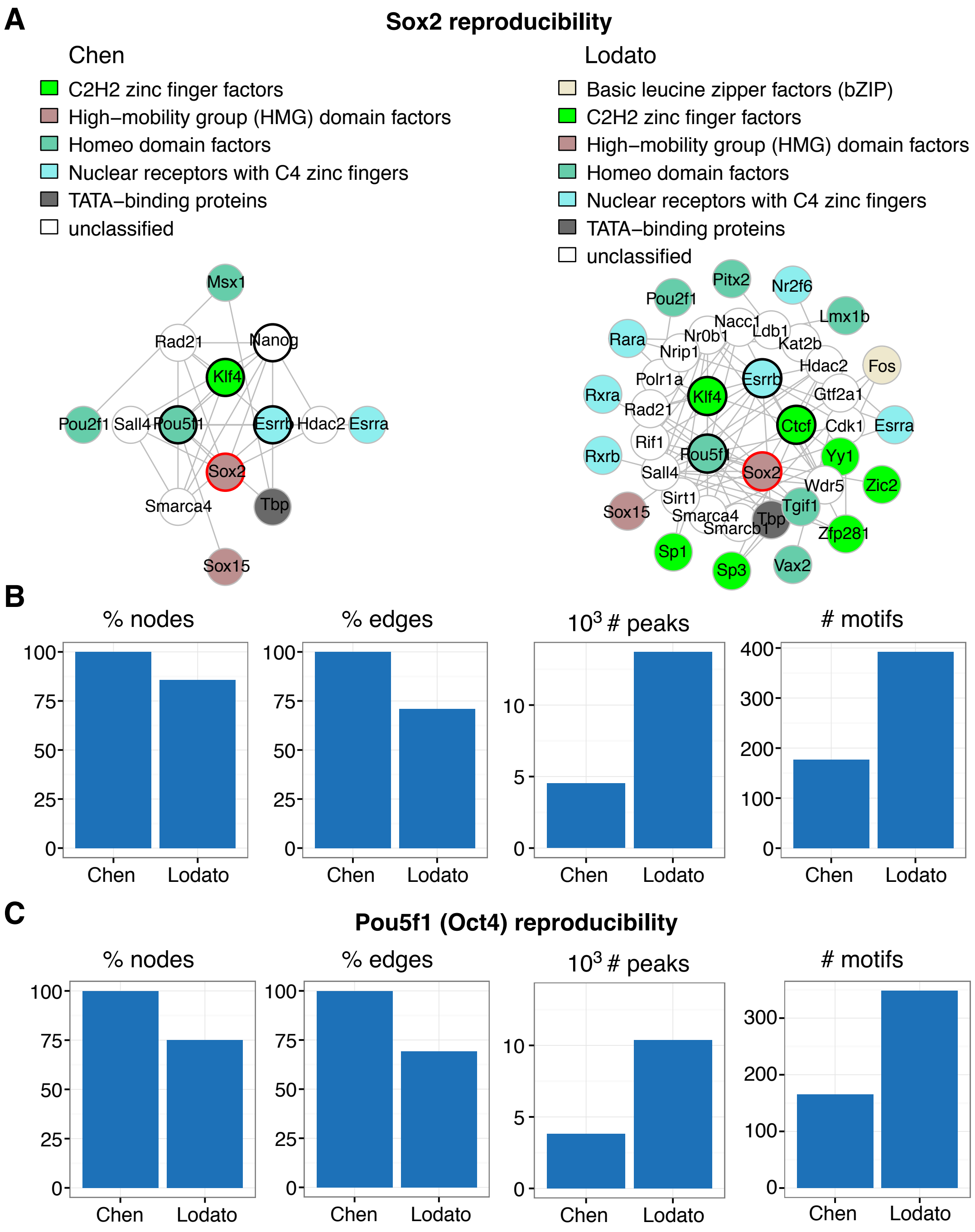

Supplementary Figure 8. The Esrrb and Sox2 regulatory networks are functionally interdependent, and they auto-regulate Esrrb and Sox2, as well as other genes of the TRMs. (A) The Esrrb and Sox2 TRMs, and their common nodes. (B) Change in gene expression of the genes within both regulatory networks in ESCs, where Sox2, Esrrb or Sox2/Esrrb (SoES) are knocked down. 'GFP' is a GFP-specific shRNA used as a control. Microarray data is from GSE34170. Significance (t-test) is indicated for significantly changing gene sets. (C) Heatmaps showing the fold-change of the genes from the two TRMs in the Esrrb knockdown (left) and the Sox2 knockdown (right).

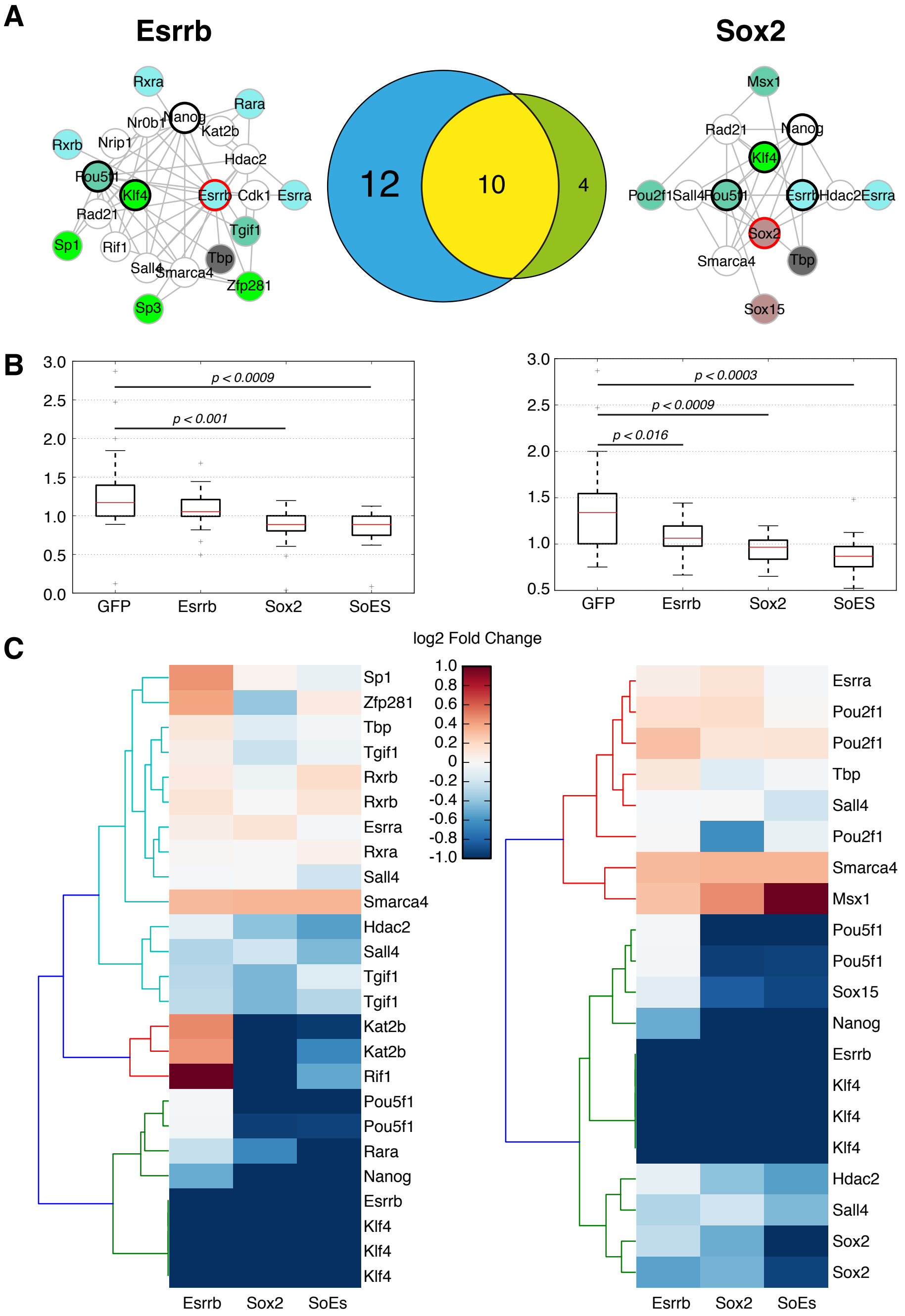

Supplementary Figure 9. Gene Ontology (Biological Process) terms related to "stem cell" in all the ESC modules. The p-value is indicated using a colour scale (darker colours indicating greater significance). Grey indicates missing values (i.e. terms not enriched for that module at $p<0.05$). Terms and modules are clustered using *Euclidean* distance and complete linkage.

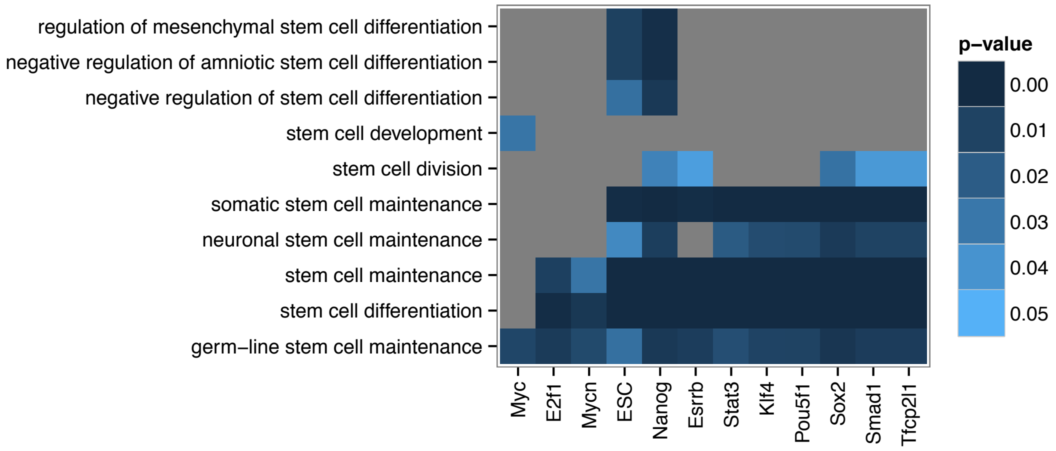

Supplementary Figure 10. Individual TRMs reconstructed for 9 TFs in HSCs.

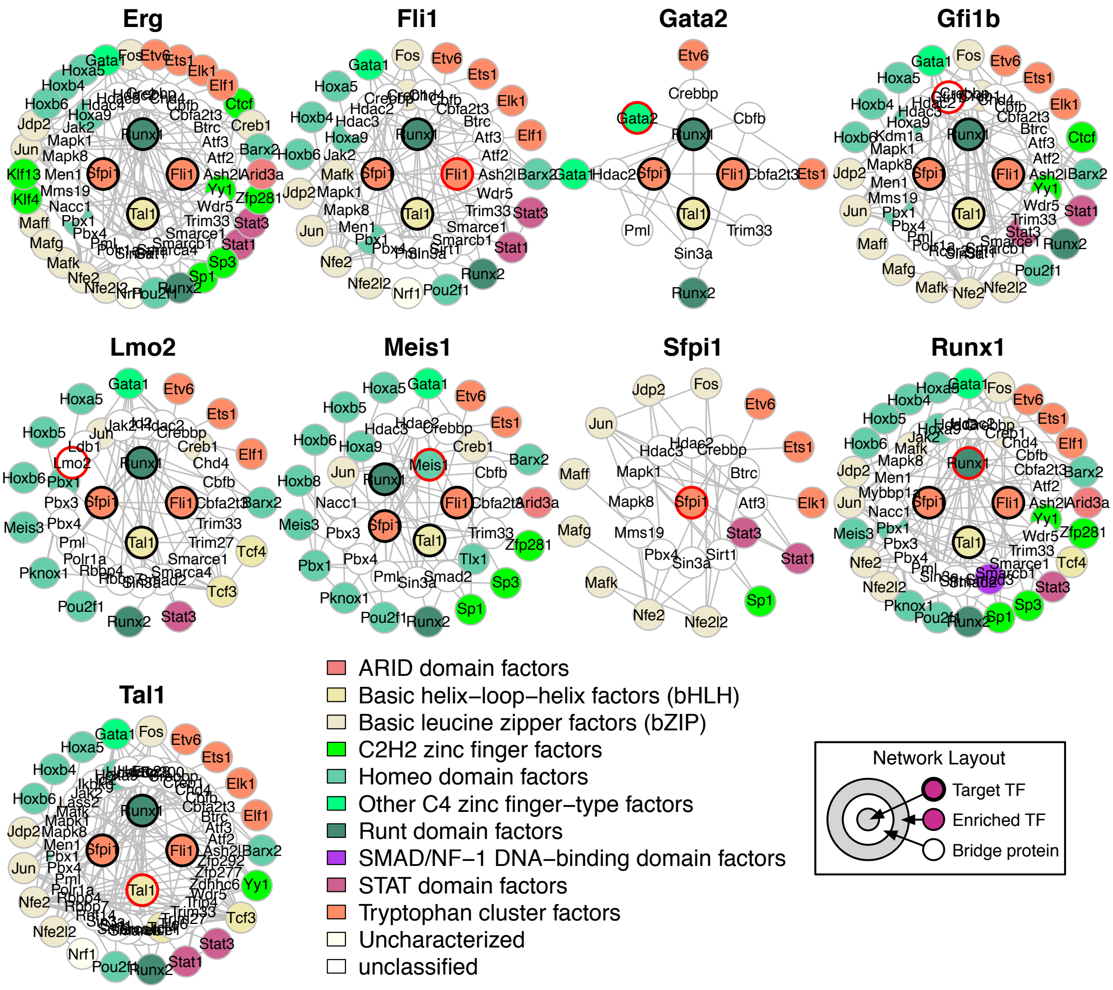

Supplementary Figure 11. Gene Ontology (Biological Process) terms related to "hemopoietic" and "stem cell" in all HSC modules. The p-value is indicated using a color scale (darker colors indicating greater significance). Grey indicates missing values (i.e. terms not enriched for that module at p<0.05). Terms and modules are clustered using *Euclidean* distance and complete linkage.

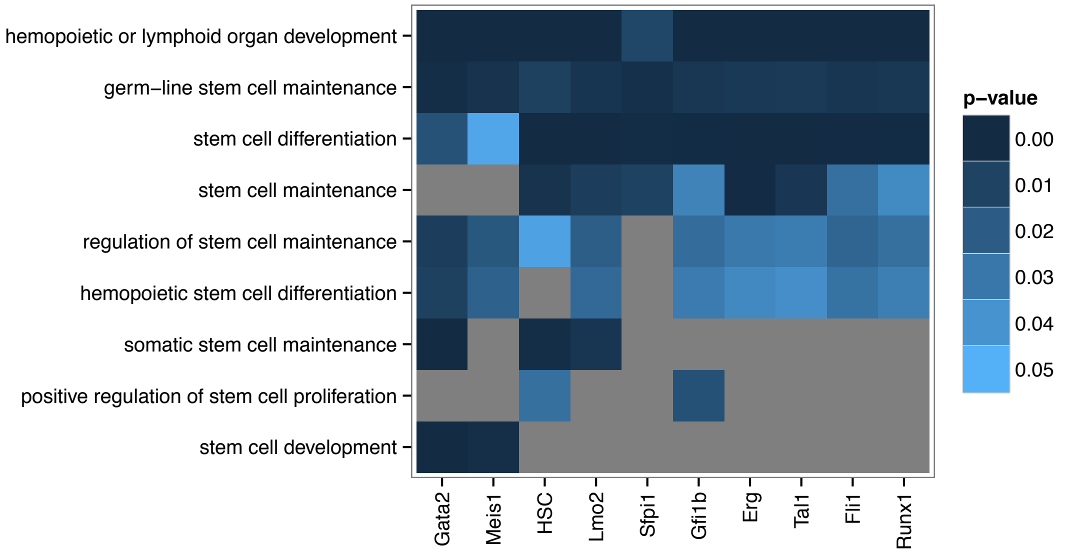

Supplementary Figure 12. TRMs identified in (A) TSCs for Cdx2, (B) NPCs for Sox2, and (C) during CD4+ T cell development for Gata3. Although the NPC dataset contained also data for Pou3f2, and the TSC dataset contained data for Elf5 and Eomes, these TFs could not be found in the PPI network and therefore the corresponding TRMs could not predicted.

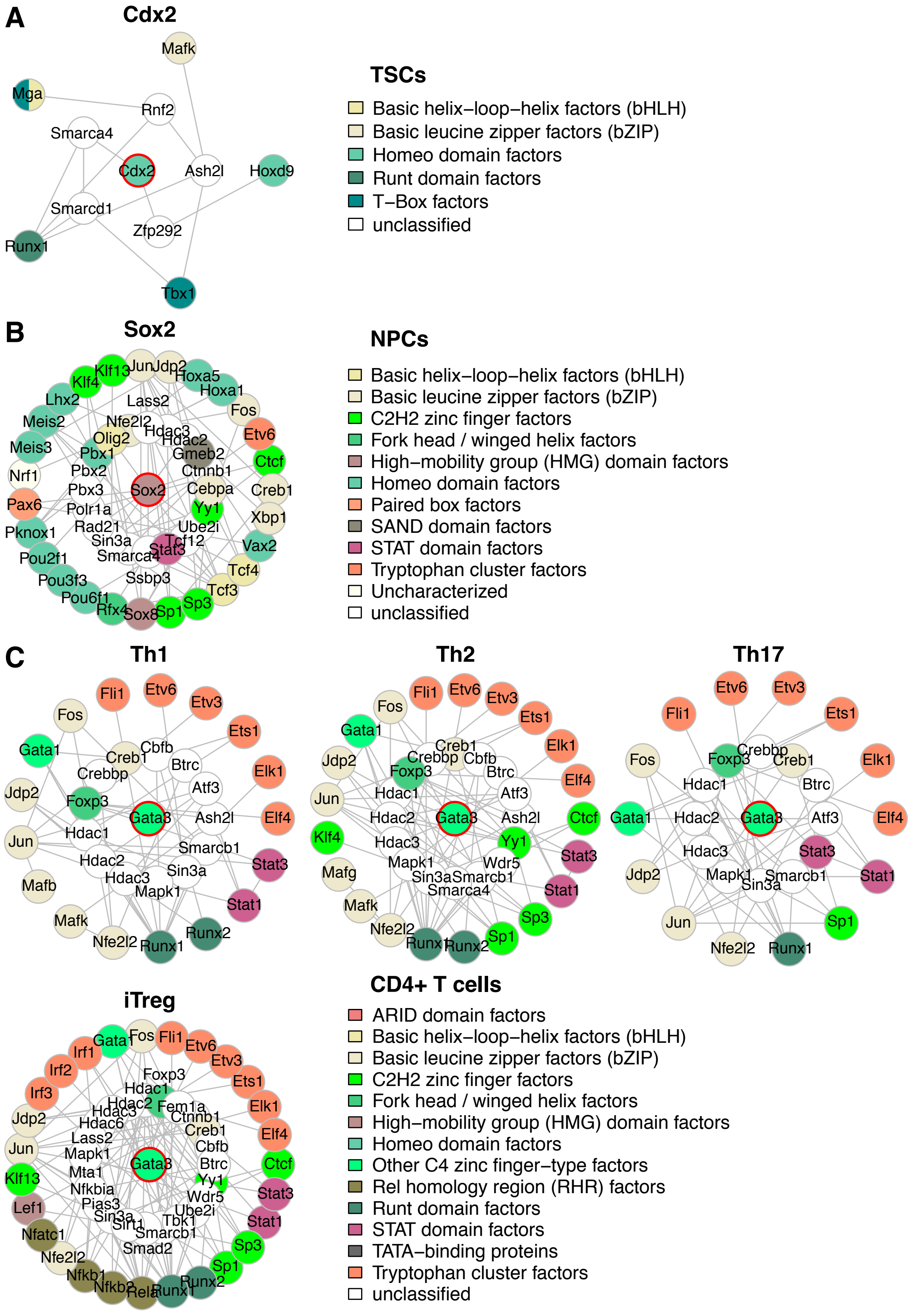

Supplementary Figure 13. Combined network for CD4$^+$ T cells. The different TRMs identified in CD4$^+$ T cells were combined and the nodes coloured according to their presence in one or more CD4$^+$ T cell type-specific TRMs.

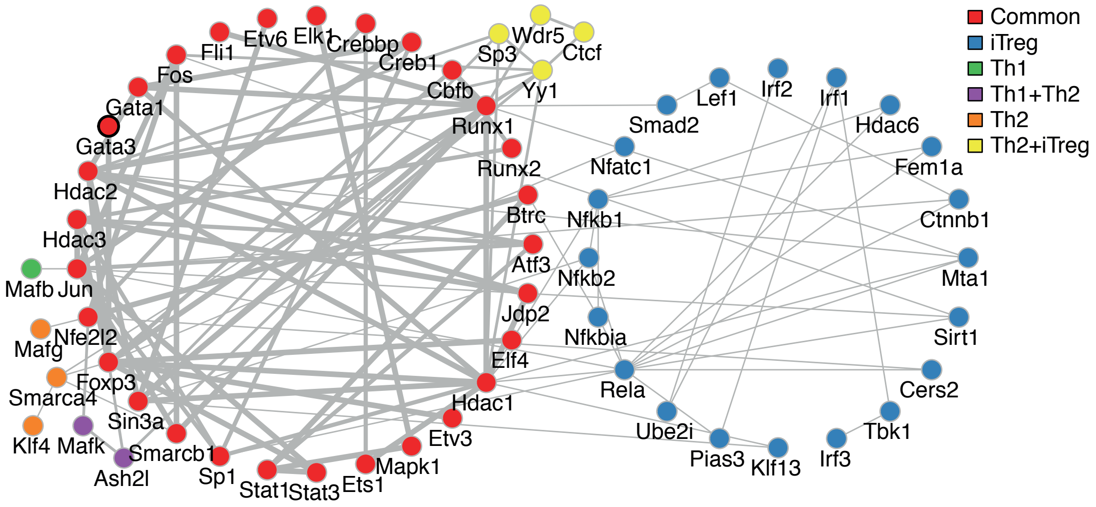

Supplement: Supplementary Data [file supp_gkt913_nar-01249-met-n-2013-File006.pdf]
